# Book Review: *Further understanding of the human machine. The road to bioengineering*

**DOI:** 10.1186/s12938-017-0382-3

**Published:** 2017-08-02

**Authors:** Alvin Wald

**Affiliations:** Columbia University, New York, US

## Abstract

Max E. Valentinuzzi (editor)

*Further understanding of the human machine. The road to bioengineering*

Series on bioengineering and biomedical engineering, volume 7.

Singapore/New Jersey: World Scientific Publishing Co. Pte. Ltd.

pp. xxxi+546. ISBN 9789813147256.

This is not an easy book to review. Its scope is wide and deep. So this review will not be in the traditional style. Instead, I will simply present a brief overview of the contents along with my own opinions of its strengths and weaknesses. First, I must state that for some 40 years I have been a collaborator and good friend of the editor of this book, Max E. Valentinuzzi. Although we live on different continents, we are contemporaries with similar backgrounds and many interests in common, particularly an historical appreciation of biomedical engineering and a recognition of the vast scope of this discipline. For a more complete view of his philosophy (and perhaps a bit of my own), I suggest the biosketch that you can find at: https://biomedical-engineering-online.biomedcentral.com/articles/10.1186/1475-925X-11-1.

This volume is and is not both a textbook and a reference source. It is a follow-up or sequel to Professor Valentinuzzi’s 2004 book in this same series (volume 4) “understanding the human machine: a primer to bioengineering.” The current work revises, expands, and extrapolates this earlier incarnation. As such, it covers the traditional aspects of bioengineering, but with what I would call a unique perspective. The organization of this book is familiar, chapters on the various physiological systems such as renal, cardiac and respiratory. The book can be happily and productively used by students to learn basics and by practicing biomedical engineers to refresh knowledge. Both types of users would be amply rewarded in following this book. However, I must caution the casual reader that the scope of the material found within is almost overwhelming. The bulk of this work would be most appreciated by those with a broad knowledge of our cultural and scientific universe.

I must also point out that this volume as well as my review are based on a somewhat old-fashioned approach to biomedical engineering. When Professor Valentinuzzi (and I) started out in the field, the emphasis was on using the principles of the various engineering disciplines as applied to the physiological sciences. Much has changed since then. Nowadays, many biomedical engineering programs and activities include such areas as nanotechnology, tissue and cell engineering, genomics, and proteomics.

This book’s unique perspective includes uncountable references for each of the 14 chapters. Overall, there must be literally hundreds of references cited. Furthermore, there are numerous referrals to all types of material, from Walt Whitman to Stephen Hawking. This aspect of the work was particularly appealing to me. However, being a former editor myself, I must say that, by itself, the text requires some effort to appreciate, as the contributors are not native speakers or writers of English.

For one who has savored and loved books since my early childhood, I was much impressed by its heft when this volume arrive. I actually put it on a scale, where it registered a robust 2.25 lb. The cover and binding were substantial. The paper of the pages was thick with an ample feel, and the print size was large with appreciable line spacing surrounded with lots of white borders. There are many illustrations, some hand-drawn with color. There is an index for all of the technical topics covered, but unhappily it does not include the references to the many interesting asides and quotations. Such an inclusion would probably have added more pages than allowable. I would call this book a volume to behold and to hold.

Overall, a very worthwhile addition to the biomedical engineering literature. Or perhaps as a starter volume to a personal library or as a special gift to a student or friend.

